# Cytological Effects of Serum Isolated from Polytraumatized Patients on Human Bone Marrow-Derived Mesenchymal Stem Cells

**DOI:** 10.1155/2021/2612480

**Published:** 2021-11-28

**Authors:** Yazhou Long, Katrin Bundkirchen, Pascal Gräff, Christian Krettek, Sandra Noack, Claudia Neunaber

**Affiliations:** Trauma Department, Hannover Medical School, 30625 Hannover, Germany

## Abstract

Due to their immunomodulatory and regenerative capacity, human bone marrow-derived mesenchymal stem cells (hBMSCs) are promising in the treatment of patients suffering from polytrauma. However, few studies look at the effects of sera from polytraumatized patients on hBMSCs. The aim of this study was to explore changes in hBMSC properties in response to serum from polytrauma patients taken at different time points after the trauma incident. For this, sera from 84 patients with polytrauma (collected between 2010 and 2020 in our department) were used. In order to test the differential influence on hBMSC, sera from the 1^st^ (D1), 5^th^ (D5), and 10^th^ day (D10) after polytrauma were pooled, respectively. As a control, sera from three healthy donors (HS), matched with respect to age and gender to the polytrauma group, were collected. Furthermore, hBMSCs from four healthy donors were used in the experiments. The pooled sera of HS, D1, D5, and D10 were analyzed by multicytokine array for pro-/anti-inflammatory cytokines. Furthermore, the influence of the different sera on hBMSCs with respect to cell proliferation, colony forming unit-fibroblast (CFU-F) assay, cell viability, cytotoxicity, cell migration, and osteogenic and chondrogenic differentiation was analyzed. The results showed that D5 serum significantly reduced hBMSC cell proliferation capacity compared with HS and increased the proportion of dead cells compared with D1. However, the frequency of CFU-F was not reduced in polytrauma groups compared with HS, as well as the other parameters. The serological effect of polytrauma on hBMSCs was related to the time after trauma. It is disadvantageous to use BMSCs in polytraumatized patients at least until the fifth day after polytrauma as obvious cytological changes could be found at that time point. However, it is promising to use hBMSCs to treat polytrauma after five days, combined with the concept of “Damage Control Orthopedics” (DCO).

## 1. Introduction

Polytrauma is not a mere addition of multiple injuries; instead, it has significant effects on the entire body function and changes in pathophysiology. People affected by this kind of injury often suffer from serious disabilities and, in extreme cases, lose their lives. Therefore, polytrauma is a leading cause of death and disability worldwide [1, 2]. A classification for polytrauma is important for proper and accurate benchmarking of care and results [3]. The most widely accepted classification system for polytrauma is the Injury Severity Score (ISS). For its calculation, first, the Abbreviated Injury Scale (AIS), which classifies each injury by body region on a 6-point scale, is used. Next, the ISS is derived from the AIS by calculating the sum of the squares of the AIS scores of the three most severely injured body regions [4, 5]. The internationally accepted criterion for serious polytrauma is an ISS ≥ 16 [6, 7]. The new “Berlin definition” states that polytrauma is defined by an AIS ≥ 3 for two or more different body regions and one or more additional variables from five physiologic parameters [8].

Polytrauma induces serious multisite damage and can even result in multiple organ dysfunction syndrome (MODS) and sepsis [8, 9]. The nature of polytraumatic inflammatory response is the body's physiological immune response to damage and microorganisms [10–12]. Trauma triggers the production of different inflammatory mediators and associated peptides. Tumor necrosis factor-*α* (TNF-*α*), interleukin- (IL-) 1, IL-6, IL-8, and IL-10 can be secreted from monocytes/macrophages [12, 13]. Inflammatory cascade activation plays a major role in the development of immune dysfunction and MODS following polytrauma [14].

Mesenchymal stem cells (MSCs) are defined as nonhematopoietic multipotent stromal stem cells which are characterized by their ability for self-renewal and to differentiate into adipocytes (fat), osteoblasts (bone), and chondrocytes (cartilage) [15]. Human bone marrow-derived mesenchymal stem cells (hBMSCs), as one of the most studied cells in bone and cartilage tissue engineering, show good osteogenic and cartilage potential [16]. MSCs possess immunomodulatory properties that can be activated by signalling molecules such as interferon gamma (IFN-*γ*), TNF-*α*, and IL-1*α*/*β* [17, 18]. Distinctive chemokines induce MSC migration to the site of injury, for instance, stromal cell-derived factor 1 *α* (SDF-1*α*) [19].

As a defect of bone and cartilage is often a common complication after trauma [20], MSCs, which can be induced to differentiate into osteoblasts and chondrocytes *in vitro*, are promising cells to treat these defects [21–23]. Therefore, a promising solution is called the MSC-based bone regeneration [24], where polymer scaffolds seeded with MSCs are used to induce differentiation of MSCs into osteoblasts *in vivo*. Experimental studies have shown that good results have already been achieved [24–26]. Chondrocytes can be obtained by culturing MSCs in a special differentiation medium using classic pellet culture methods [27], and cartilage can also be generated with the help of tissue engineering techniques using 3D cell scaffolds (e.g., hydrogels [28] and electrospun scaffolds [29]) *in vitro*.

Improving the treatment of polytrauma and strengthening posttraumatic care are a difficult challenge for the world. The rationale for using MSCs in polytrauma patients is their anti-inflammatory immune regulation and tissue regeneration capacity [30]. However, it is known that the complex changes of serum cytokines after polytrauma [31] exert a complex impact on the characteristics and properties of MSCs [30, 32]. Therefore, the effects of inflammatory mediators on MSCs present in the blood of multiple trauma patients at different times after trauma should be further investigated. So far, there have been only a few studies on this topic [30, 33]; due to our knowledge, no studies using large number of serum samples exist in which the influence of sera from different time points posttrauma was investigated on MSC properties. Therefore, the aim of this study was to find out whether serum from different days after polytrauma (D1, D5, and D10) has a negative influence on cell survival and the ability of hBMSCs to migrate, proliferate, and differentiate. In this study, it was hypothesized that higher cytokine levels in D1 and D5 sera may lead to reduced proliferation of hBMSCs, which can lead to a decrease of CFU-F colonies and an obvious migration of hBMSCs compared with HS and D10 sera. In addition, it was postulated that osteogenic and/or chondrogenic differentiation may be significantly inhibited due to the high cytokine levels of D1 or D5 sera.

## 2. Materials and Methods

### 2.1. Source of Serum and Cells

The serum of polytrauma patients (ISS ≥ 16) was obtained from the Polytrauma Serum Bank of the Trauma Department of Hannover Medical School (year: 2010-2020). The study protocol and sample donation were approved by the local ethical review committee (Hannover Medical School No. 4980). 84 patients with polytrauma (median of ISS = 34, interquartile range: 27-43.5) were used as donors, and the sera from the 1^st^ day, 5^th^ day, and 10^th^ day after trauma were pooled to ensure sufficient amounts. As control, three healthy donor sera were matched and pooled (HS) according to the age and gender ratio of patients with polytrauma. For the experiments, four healthy hBMSC donors were chosen from our cell bank of human bone marrow-derived mesenchymal stem cells. hBMSC donation protocol and sample donation were approved by the local ethical review committee (Hannover Medical School No. 2562). BMSC selection was based on the following criteria: no history of smoking, alcohol, allergies, tumors, and routine medication. Written informed consent was obtained from all donors prior to inclusion into the studies, and it was ensured that the study protocol and process of sample donation for the studies complied with the Declaration of Helsinki. In Table 1, the age distribution, gender, and ISS score of polytraumatized patients (PT), healthy serum donors (HS), and hBMSC donors can be seen.

### 2.2. Multicytokine Array

Semiquantitative detection of multicytokines was performed by Tebu-bio (Paris, France) using the Human Cytokine Antibody Array Kit (Ref: AAH-CYT-G5-8, RayBio®) [34] with pooled serum as duplicate from HS, D1, D5, and D10. The change of cytokine concentration is shown as heat map which was first calculated by normalized fluorescence intensity (NFI) and then normalized by standard score (*Z*-score) and analyzed by fold change relative to healthy serum (FCRH).

### 2.3. Cell Culture and Passaging

Stored hBMSCs were thawed and cultured with hBMSC normal growth medium, which contained 10% fetal calf serum (FCS, #S0615, Bio-cell), 1% penicillin/streptomycin (#A2212, Biochrom), 2.5% hydroxyethyl piperazineethanesulfonic (HEPES, #L1613, Biochrom), 86.5% Dulbecco's modified Eagle medium (DMEM, #FG0415, Bio-cell), and 100 ng/*μ*l human-fibroblast growth factor-2 (h-FGF-2, #100-18B, PeproTech) in cell culture flasks at 37°C and 5% CO_2_. Upon reaching a confluency of 80-90%, cells were harvested by using 0.05% Trypsin/EDTA (0.5%/0.2% *w*/*v*, #L1825, Biochrom) solution and counted by flow cytometry (Attune NxT, Thermo Fisher Scientific, USA). In order to ensure that there were enough cells for the experiments, the cells were expanded and all experiments were performed using hBMSCs in passage 3 (P_3_).

### 2.4. Colony Forming Unit-Fibroblast (CFU-F) Assay

250 cells were seeded in 6-well plates using media corresponding to each group (10% HS/D1/D5/D10). Total medium change with corresponding media was performed on day 3 and day 7 after seeding. The CFU-F assay was stopped on day 10 by fixating the cells with 2 ml cold methanol and staining with 1% aqueous crystal violet at room temperature for 30 minutes before performing macroscopically visible colony counting. The results are shown as percentage of the number of colonies per 250 cells seeded.

### 2.5. Cell Proliferation Experiments and Live and Dead Assay

30,000 cells were seeded in each well of a six-well plate and incubated with corresponding media for each group (10% HS/D1/D5/D10) for 72 hours at 37°C and 5% CO_2_. After that, the cells were detached with 0.05% Trypsin/EDTA and stained with Fixable Viability Dye eFluor™ 450 (#650863, Thermo Fisher Scientific) and evaluated by flow cytometry (Attune NxT Flow Cytometer; Thermo Fisher Scientific, USA). The total cell number and percentage of dead cells in each well were calculated by the Invitrogen™ Attune™ NxT Software (Attune; Thermo Fisher Scientific, USA) at the same time.

### 2.6. WST Assay

To assess cell viability/proliferation and cytotoxicity, a WST assay was performed. WST-1 (#5015944001, Roche) is a tetrazolium salt which forms formazan in the presence of metabolically active cells. 100 *μ*l hBMSC suspension (8000 cells) was added to each well in a 96-well plate, and each experimental group was tested in three replicates. After incubation for 24 hours at 37°C and 5% CO_2_ with the corresponding media, 10 *μ*l WST-1 reagent was added to each well. After 30 minutes, 1 hour, 2 hours, and 4 hours, the absorbance at 450 nm of each well was measured using an Epoch reader (Epoch, USA) and analyzed by Gen 5™ software (BioTek, Winooski, VT, USA), to quantify the cell activity. The absorbance at 630 nm was measured for correction of unspecific background absorption.

### 2.7. Transwell Experiments

600 *μ*l of cell culture medium containing 10% human serum from one of the serum groups (HS, D1, D5, and D10) was added into each well of a 24-well plate (#662160, CellStar) with three duplicates for each serum group. Then, 1 × 10^5^ cells were resuspended in 100 *μ*l serum-free media and pipetted to the upper chamber of each tissue culture insert (TC, 24-well plate, pore 8 *μ*m, Sarstedt, Germany). These TC inserts were then placed into the wells containing the 10% human serum as lower well. After cultivation at 37°C and 5% CO_2_ for 12 hours, the medium from all upper and lower chambers was discarded. 600 *μ*l 0.05% Trypsin/EDTA solution was pipetted to the lower well of each TC insert, respectively, and for liquid balance purposes, 100 *μ*l 0.05% Trypsin/EDTA solution was pipetted to the upper chamber. After 8 minutes of incubation at 37°C and 5% CO_2_, the cells that migrated to the lower well were counted separately. The percentage of cells that had migrated from the upper to the lower chamber was calculated with the following formula:

(1) $$\begin{array}{c} \text{Migration}\left[\%\right]=\frac{\text{Number of migrated cells}}{1\times 10^{5}\text{ cells}}\times 100\%. \end{array}$$

### 2.8. Scratch Assay

30,000 cells from each group, which were pretreated for one passage with the corresponding sera (HS, D1, D5, and D10), were resuspended in 70 *μ*l serum-free medium. *ibidi* inserts (#81176, ibidi, Germany) were placed in a 6-well plate, and the cell suspension was added to corresponding *ibidi* inserts. The plates were transferred into an incubator at 37°C and 5% CO_2_ for 6-8 hours until the seeded cells formed a monolayer. Then, the *ibidi* inserts were removed from the 6-well plates and 2 ml of serum-free media was added to each well after the cells and media had been rinsed with PBS. After this, a defined scratch in the middle of the cells was visible after the removal of the inserts. Pictures of each *scratch* were taken at 0, 4, 8, and 24 hours under an optical microscope (CKX41 Olympus, Tokyo, Japan) with a magnification of 40x. Photoshop software (version 21.0.2, Adobe Company, USA) was used to merge the images, and the scale of the crop area size was set to 2000 pixels × 8000 pixels with a picture resolution of 72 ppi. Microsoft PowerPoint Software (version 16.37, Microsoft® Office 365 for MAC, Microsoft Corporation, Washington, USA) was used to define and scribe the boundary of cell migration and color the region free of migrated cells in the gap. Afterwards, the images were analyzed by a self-written tool from a member of the laboratory relying on the OpenCV library version 4.1.0 [35] for image processing. The nonmigrated proportion was shown by the percentage of black pixels, and 100% nonmigrated region was obtained at the initial 0 hour. Then, the cell migration area ratio at each time point was obtained from the decreased proportion of the area of interest.

### 2.9. Osteogenic Differentiation

15 × 10^4^ cells were resuspended in 2 ml 10% FCS normal growth media per well in 6-well plates. After cultivation in an incubator at 37°C and 5% CO_2_ for 24 hours, the media were changed to osteogenic differentiation media consisting of DMEM (#FG0415, Bio-cell), which contained 10% serum (HS, D1, D5, D10, or FCS) and was supplemented with 20 mM HEPES (#L1613, Biochrom), 1% penicillin/streptomycin (#A2212, Biochrom), 100 nM dexamethasone (#D4902, Sigma), 50 *μ*M ascorbate-2-phosphate (#A8960, Sigma), and 3 mM di-Natriumhydrogenphosphat-Dihydrat (#106508, Merck). The osteogenic differentiation should be performed over 28 days with medium change every seven days and was stopped by fixing the cells with 4% formalin solution. Staining was performed with 0.5% alizarin red solution (pH = 4.5) protected from light for 10 minutes at room temperature.

Pictures at 40x magnification were taken along the central axis of the wells using a CKX41 Olympus microscope. The images were analyzed by a self-written tool from a member of the laboratory relying on the OpenCV library version 4.1.0 [35]. The calcium deposit proportion was calculated from the percentage of nonblack pixels.

For a second measurement to evaluate the osteogenesis, the stained cells were incubated in 10% cetylpyridinium chloride solution (#8400080025, Sigma-Aldrich) adjusted to a pH of 7 at room temperature for six hours until all red color had completely dissolved. The relative content of alizarin red was then measured by determining its light absorbance at 600 nm using an Epoch reader (Gene 5, BioTek, Winooski, VT, USA).

### 2.10. Chondrogenic Differentiation

For each group, 2.5 × 10^5^ cells were resuspended in 2 ml of normal growth media containing 10% FCS in a 15 ml falcon tube (#188271, CellStar) and centrifuged for 10 minutes at 200 × *g* and room temperature to form a pellet. With the lid slightly open to allow air circulation, the cell pellet was cultured in an incubator at 37°C and 5% CO_2_ for 24 hours, and then, the medium was replaced by group-specific chondrogenic differentiation medium of DMEM (#FG0435, Bio-cell), which contained 10% serum (HS, D1, D5, D10, FCS, and serum-free) and was supplemented with 20 mM HEPES (#L1613, Biochrom), 1% penicillin/streptomycin (#A2212, Biochrom), 100 nM dexamethasone (#D4902, Sigma), 170 *μ*M ascorbate-2-phosphate (#A8960, Sigma), 1 mM Natriumpyruvat (#L0473, Biochrom), 350 *μ*M proline (#1713.1, Roth), 10 *μ*l/ml insulin (#I0516, Sigma), and 1 *μ*l/ml TGF-beta-3 (#100-36E, PeproTech). A medium change was performed every seven days for planned 28 days. Stopping the experiment was performed by fixation of the cells with 4% formalin. After fixation, the chondrogenic pellets were embedded in Tissue-Tek® O.C.T.™ Compound and wrapped in aluminum foil. The samples were frozen by placing the foil in liquid nitrogen. Afterwards, the pellets were stored at -20°C until further use.

The chondrogenic pellets were cut to slices with a thickness of 5 *μ*m using a microtome cryostat (Microm HM 500 OM, Leica) and placed on microscope slides (SuperFrost Plus, Thermo Scientific). The slides were stained by 0.1% safranin O solution for 15 minutes at room temperature and were embedded with Vitro-Clud® (R. Langenbrinck GmbH) after drying.

Pictures of chondrogenesis were taken using a microscope (BX41 Olympus) at 40x magnification. The pictures were analyzed by a self-written tool from a member of the laboratory relying on the OpenCV library version 4.1.0 [35]. The proportion of the histological specimen containing glycosaminoglycans was calculated from the percentage of nonblack pixels.

### 2.11. Data Analysis and Statistics

Statistical data analysis was performed by using the SPSS Statistics® program (Version 26, IBM SPSS Statistics Corp., New York, NY), while figures were created using Prism 8 software (Version 8.4.0 for Mac OS, GraphPad Company, Santiago) and a heat map was created by Morpheus (Cambridge, Massachusetts, USA) [36]. First, the Shapiro-Wilk test was used to determine whether each set of data met the normal distribution, where a *p* value greater than 0.05 was indicative of normal distribution. The normally distributed data were analyzed by one-way ANOVA and the Tukey test, and the nonparametric data were analyzed by the Kruskal-Wallis test with Bonferroni correction. Descriptive *p* values or adjusted *p* values less than or equal to 0.05 were considered statistically significant.

## 3. Results

### 3.1. Multicytokine Array

**Figure 1:**
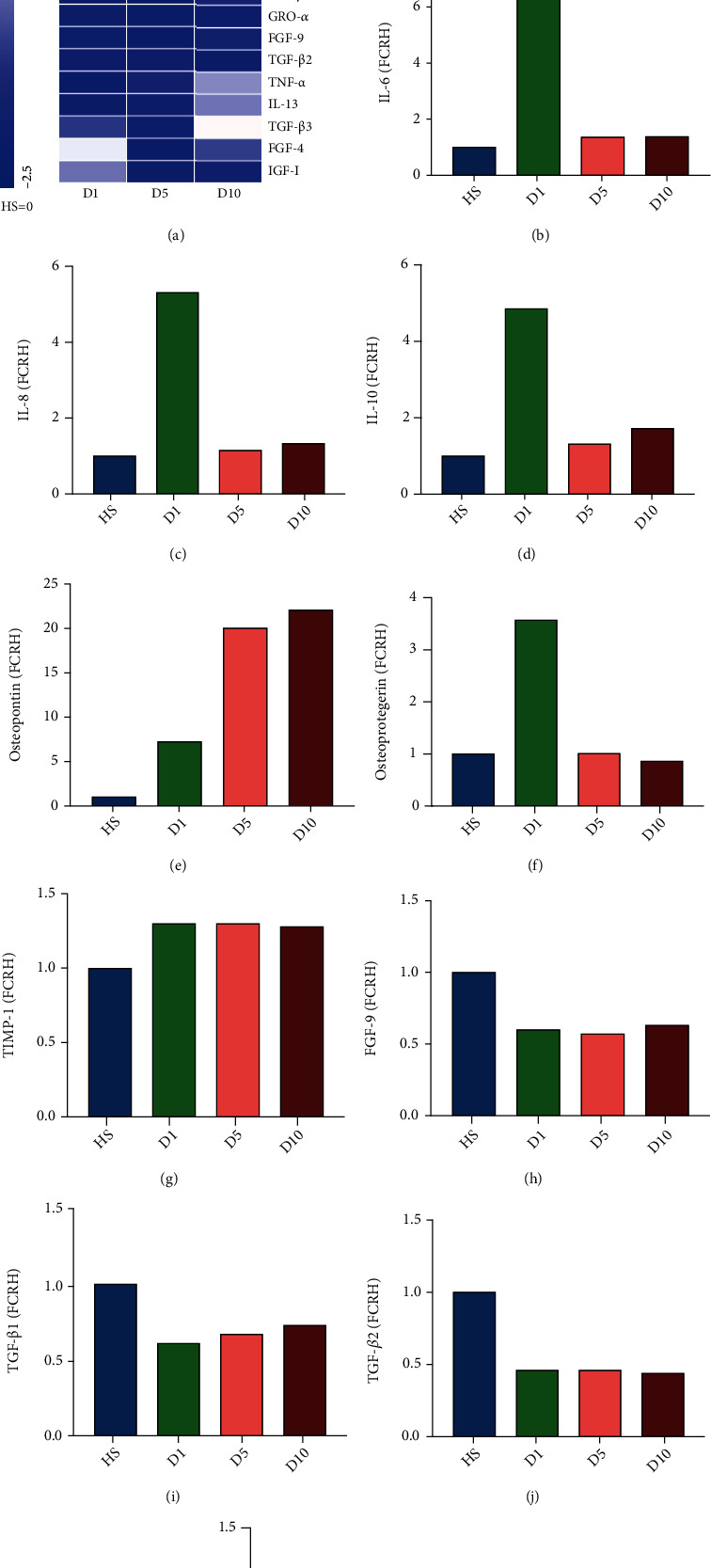
Results of the multicytokine array. (a) Heat map depicting the changes in cytokine levels in pooled serum from patients at day 1 (D1), day 5 (D5), and day 10 (D10) after polytrauma relative to cytokine levels in serum from healthy controls. The data are normalized by *Z*-score (HS = 0) and analyzed by fold change relative to healthy serum (FCRH). A *Z*-score of 0 is represented by white color, a *Z*-score of 2.5 by red color, and a *Z*-score of -2.5 by blue color. *Z*-scores in between these values are depicted by less saturated colors of the same color palette. Cluster analysis was performed, and the cytokines were ordered accordingly. Some cytokines have an acute peak on the first day after trauma, and some cytokines peak on the 5^th^ or 10^th^ day after trauma, but compared to HS, more than half of the cytokine levels decreased after trauma. The proinflammatory cytokines (b) interleukin- (IL-) 6 and (c) IL-8 as well as the anti-inflammatory cytokine (d) IL-10 were approximately four times higher at D1 in comparison to HS and two or three times higher when compared to D5 and D10. (e) Osteopontin was increased in the serum from all time points postpolytrauma as compared to serum from healthy donors. The increase in D1, D5, and D10 relative to HS was from six to twenty times. (f) Osteoprotegerin was higher only one day after polytrauma in comparison to HS with a fold change for about three times. (g) Tissue inhibitor of metalloproteinase (TIMP-1) was increased at all time points after polytrauma in comparison to serum from healthy controls. (h) Fibroblast growth factor 9 (FGF-9) was decreased in the polytrauma sera as compared to the HS.(i–k) Transforming growth factor-beta 1 (TGF-*β*1), TGF-*β*2, and TGF-*β*3 were all decreased in polytrauma serum at D1 and D5 as compared to healthy serum (*n* = 1, pooled samples out of 84 donors).

Since the levels of cytokines were measured twice in pooled serum samples, the results reflect the overall difference, and no statistical analysis was performed. Only the cytokines related to this experiment are shown in the main manuscript. Compared with the healthy serum, the red-colored cytokines showed an increase after trauma whereas the blue-colored cytokines were decreased (Figure 1(a)).

#### 3.1.1. Pro- and Anti-Inflammatory Cytokines

The level of the proinflammatory factors IL-6 (Figure 1(b)) and IL-8 (Figure 1(c)) as well as the level of the anti-inflammatory cytokine IL-10 (Figure 1(d)) showed a four times fold short-term increase on the first day after polytrauma compared to HS and decreased to two or three times higher when compared to D5 and D10. Five and ten days after the trauma incident, the level of IL-6, IL-8, and IL-10 had declined to the same level that was found in the HS group.

#### 3.1.2. Bone Metabolism

The level of osteopontin (Figure 1(e)) gradually increased over the first ten days after polytrauma compared to the HS group. Osteoprotegerin (Figure 1(f)) increased on the first day after trauma and decreased on D5 and D10 back to serum concentration which was also seen in the HS group. Tissue inhibitor of metallopeptidase-1 (TIMP-1, Figure 1(g)) concentration was increased in the first ten days after trauma. The serum concentration of fibroblast growth factor 9 (FGF-9, Figure 1(h)) decreased directly after trauma, and the concentration remains lower over the first 10 days compared to the HS group.

#### 3.1.3. Chondrogenesis

Among the factors that promote chondrogenic differentiation of hBMSCs, the levels of the transforming growth factor-beta (TGF-*β*) family members TGF-*β*1 (Figure 1(i)) and TGF-*β*2 (Figure 1(j)) decreased since D1 after trauma until D10 compared to the HS group. TGF-b3 (Figure 1(k)) level continued to decrease until D5; afterwards, at D10, it reached the same serum concentration as the HS group.

#### 3.1.4. Growth Factors

Serum concentration of epidermal growth factor (EGF, Figure S1-a) was four times higher at D1, two times at D5, and five time higher at D10 after polytrauma compared to the HS group. Concentration level of hepatocyte growth factor (HGF) was more than eight times higher one day after polytrauma compared to the HS group, five times higher on day five, and seven times higher on day ten (Figure S1-b). Concentration level of fibroblast growth factor 4 (FGF-4) was decreased after polytrauma compared to HS (Figure S1-c). Insulin-like growth factor-binding protein 4 (IGFBP-4) was more than two times higher on D1 and D5 after polytrauma compared to the HS group but had a downward trend at D10 which is still higher than HS (Figure S1-d).

#### 3.1.5. Migration

Among the factors that can promote chemotactic migration of MSCs, the serum concentration of insulin-like growth factor 1 (IGF-1, Figure S1-e) and growth-regulated oncogene-alpha (GRO-*α*, Figure S1-f) decreased after polytrauma as compared to the HS group. In contrast, the concentration of monocyte chemoattractant protein 1 (MCP-1, Figure S1-g) was increased in the serum of patients with polytrauma on D1 after the incidence. The serum level of vascular endothelial growth factor (VEGF) showed a one- to twofold increase from D1 to D10 compared to HS (Figure S1-h). The serum concentration of platelet-derived growth factor-BB (PDGF-BB) was increased in all groups after polytrauma compared to HS (Figure S1-i).

### 3.2. Colony Forming Unit-Fibroblast Assay

**Figure 2:**
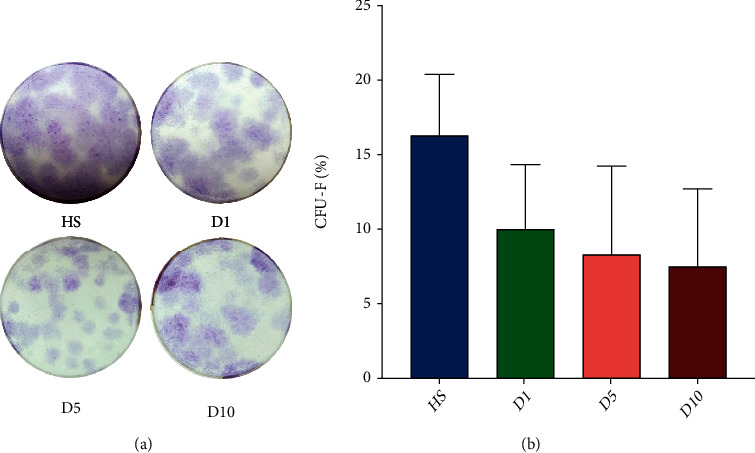
Colony forming unit-fibroblast assay (CFU-F assay). (a) Representative images of CFU-F assays from HS, D1, D5, and D10. (b) Results of the CFU-F assays obtained by seeding 250 cells/well. Calculation was performed using the following formula: CFU‐F% = colonies/250 × 100%. The colony forming ability of polytrauma groups (D1, D5, and D10) had no significant difference when compared to the HS group (*n* = 4).

Representative images of the CFU-F assay from HS, D1, D5, and D10 are shown in Figure 2(a). The data were normally distributed and met the criteria for homogeneity of variance, and one-way ANOVA and the Tukey test were used for statistical analysis. Counting of the colonies demonstrated no significantly reduced colony forming ability at D1 (10.00 ± 4.35%), D5 (8.30 ± 5.95%), and D10 (7.50 ± 5.22%) compared to the HS group (16.30 ± 4.10%, *p* > 0.05). Also, no significant differences were found between the polytrauma groups D1, D5, and D10 (Figure 2(b)). However, the blue staining area of colonies in polytrauma groups (D1, D5, and D10) was less than HS in visual (Figure 2(a)).

### 3.3. Cell Counting and Viability Measurement

**Figure 3:**
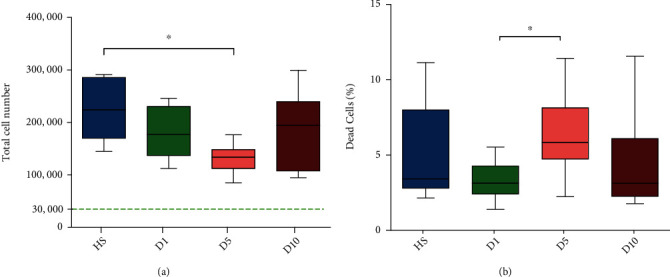
Evaluation of the proliferation capacity and viability of stem cells after cultivation in medium containing different sera (HS, D1, D5, or D10) for 72 hours. (a) Proliferation of hBMSCs treated with HS, D1, D5, or D10 serum. The green, dotted line shows the number of seeded cells (30,000 cells). Addition of serum from D5 to the culture medium resulted in a significantly lower proliferation of hBMSCs after 72 hours compared to HS. (b) Amount of dead cells after treatment with HS, D1, D5, or D10 serum. The D5 group demonstrated a significantly increased ratio of dead cells in comparison to D1. ^∗^*p* ≤ 0.05 (*n* = 4; *N* = 3).

After 72 hours of cell proliferation, hBMSCs were stained with Fixable Viability Dye eFluor™ 450 and total cell count as well as the proportion of dead cells was measured. The experimental data were nonparametric distributed, and the Kruskal-Wallis test with the Bonferroni correction was used for statistical analysis. Total cell number in the D5 group (132,071.67 ± 25,770.13 cells) was significantly reduced compared to that in the HS group (224,842.50 ± 56,999.37 cells; *p* ≤ 0.01, Figure 3(a)). No significant differences of the total cell number were observed between D1 (180,436.67 ± 47,868.25 cells, *p* > 0.05) and D10 (183,839.17 ± 73,674.91 cells, *p* > 0.05) compared to the HS group. Furthermore, the D5 group (6.56 ± 2.71%) had a significantly higher cell death ratio compared to D1 (3.37 ± 1.27%; *p* ≤ 0.05) (Figure 3(b)). In summary, the D5 group shows an obvious proliferation inhibition compared to HS as well as a higher ratio of dead cells compared to D1.

### 3.4. WST Assay

**Figure 4:**
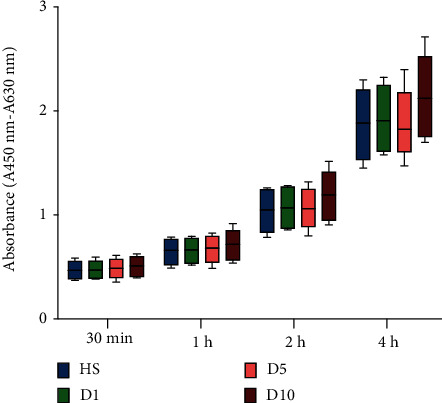
WST assay. Results were observed after cultivation of cells for 24 hours in medium containing different sera (HS, D1, D5, or D10 serum). No significant differences were observed between any of the groups at any of the time points analyzed (30 minutes, 1 hour, 2 hours, and 4 hours) (*n* = 4; *N* = 3).

24 hours after cell seeding, the cell viability and proliferation capacity were tested by WST assay after an incubation period of 30 minutes, 1 hour, 2 hours, and 4 hours (Figure 4). The experimental data were nonparametric distributed, and the Kruskal-Wallis test with the Bonferroni correction was used for statistical analysis. No significant differences in WST assay were observed among the groups at the observation point of 30 minutes (HS: 0.47 ± 0.09, D1: 0.47 ± 0.09, D5: 0.49 ± 0.10, D10: 0.51 ± 0.10, *p* > 0.05), 1 hour (HS: 0.65 ± 0.13, D1: 0.66 ± 0.12, D5: 0.67 ± 0.13, D10: 0.71 ± 0.15, *p* > 0.05), 2 hours (HS: 1.04 ± 0.21, D1: 1.07 ± 0.20, D5: 1.06 ± 0.19, D10: 1.19 ± 0.25, *p* > 0.05), and 4 hours (HS: 1.87 ± 0.36, D1: 1.93 ± 0.33, D5: 1.88 ± 0.31, D10: 2.14 ± 0.40, *p* > 0.05).

### 3.5. Migration Ability of hBMSCs

#### 3.5.1. Scratch Assay

**Figure 5:**
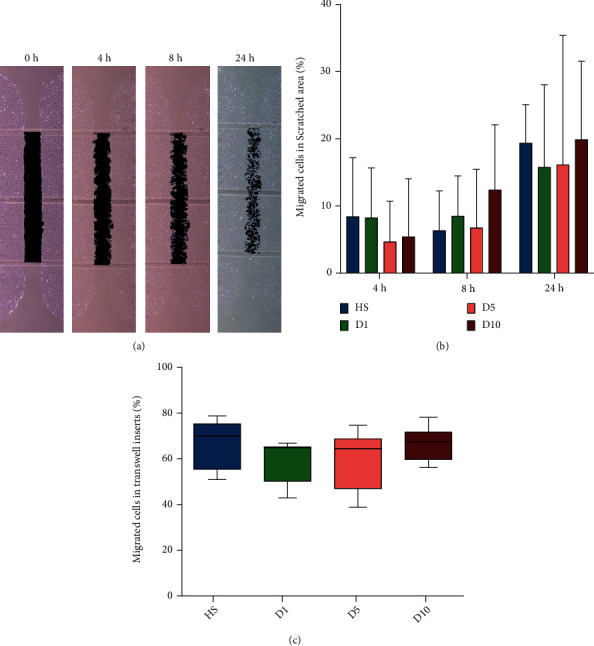
Scratch and transwell migration assay indicating the hBMSC repopulation ability. (a) Representative pictures of the migration and repopulation ability of hBMSCs over 24 hours treated with HS, D1, D5, and D10 sera in a scratch assay. The black area represents the scratched area. As time goes by, the injured area is gradually reduced and dispersed along with the migration of cells. (b) After 4, 8, and 24 hours, no significant increase in migration of the D1, D5, and D10 groups was found compared to the HS group (*n* = 4; *N* = 2). (c) In transwell migration assay, no significant differences in the ratio of migration were observed between the HS, D1, D5, and D10 groups 12 hours after seeding (*n* = 4; *N* = 3).

An example of the repopulation of the scratch with hBMSCs over different time points of the assay is shown in Figure 5(a), where the black-colored area demarks the area devoid of cells. The data were normally distributed and met the criteria for homogeneity of variance, and one-way ANOVA and Tukey test were used for statistical analysis. No significant difference was observed in the scratch assay at the different time points (4 h, *p* > 0.05; 8 h, *p* > 0.05; 24 h, *p* > 0.05) between any of the serum groups (Figure 5(b)).

#### 3.5.2. Transwell Migration Assay

The transwell migration assay data were nonparametric distributed, and the Kruskal-Wallis test with the Bonferroni correction was used for statistical analysis. Cell culture media containing the different sera from patients on D1 (60.04 ± 9.36%), D5 (60.17 ± 12.14%), and D10 (66.73 ± 7.36%) after polytrauma had no differential effect on the migration behavior of the hBMSCs in comparison to culture medium containing serum from healthy donors (67.18 ± 10.18%) and neither among groups (*p* > 0.05) (Figure 5(c)).

### 3.6. Osteogenic Differentiation

The addition of fetal calf serum (FCS) served as a positive control for the groups HS, D1, D5, or D10 for the osteogenic differentiation assay. As preliminary experiments showed that the cells detached in the presence of human serum after 21 days (data not shown), the osteogenic differentiation was stopped at day 14 instead of day 28. The experimental data were nonparametric distributed, and the Kruskal-Wallis test with the Bonferroni correction was used for statistical analysis.

**Figure 6:**
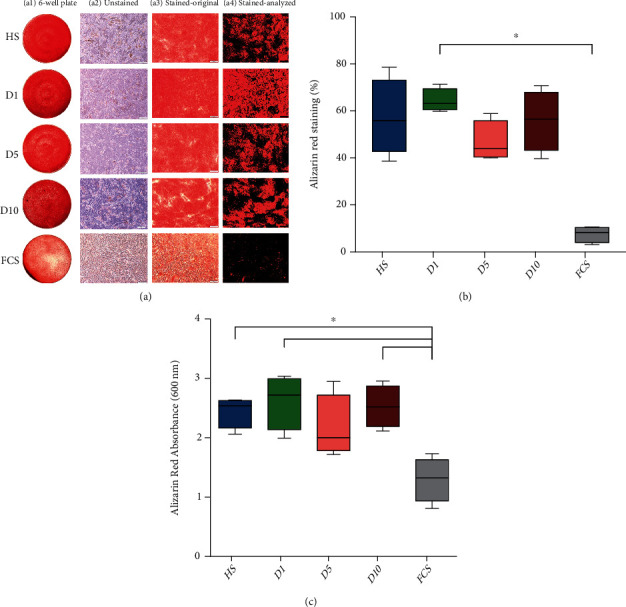
Osteogenic differentiation potential. (a) Alizarin red staining showed calcium deposits as red staining after culturing hBMSCs in osteogenic differentiation medium containing 10% serum from HS, D1, D5, D10, or FCS for 14 days. (a1) Macroscopic image of alizarin red staining. (a2) Cells prior to alizarin red staining at 40x magnification. (a3) Images showing the hBMSCs at a 40x magnification after staining with alizarin red. (a4) Analysis of alizarin red staining of calcium deposits using a tool based on OpenCV library (version 4.1.0). Areas without staining were color isolated as black areas. The pixel ratio of the area of interest (nonblack area) in comparison to the black area was obtained. In the groups containing human serum (HS, D1, D5, and D10), a larger area of the 6-well was positive for alizarin red staining than in the FCS group (*n* = 4). (b) Quantitative analysis of the amount of alizarin red staining quantified using a software tool to compare the relative areas positive for staining. D1 showed significantly more area of alizarin red staining when compared to the FCS group. ^∗^*p* ≤ 0.05 (*n* = 4). (c) Analysis of alizarin red staining indicative of osteogenic differentiation with higher concentrations of calcium deposition after 14 days of culture. HS, D1, and D10 showed significantly higher absorbance as compared to the FCS group. ^∗^*p* ≤ 0.05 (*n* = 4).

The result of the osteogenic differentiation was first quantified by image analysis stained with alizarin red (Figure 6(a)). Standardized analysis of the pictures showed that group D1 (64.35 ± 4.95%, *p* ≤ 0.01) had a higher osteogenic differentiation ability compared to the FCS group (7.52 ± 3.49%), with exception of HS (57.20 ± 16.43%, *p* > 0.05), D5 (46.74 ± 8.59%, *p* > 0.05), and D10 (55.87 ± 12.94%, *p* > 0.05). However, no significant difference was found between the D5 group compared to any of the other serum groups (*p* > 0.05) (Figure 6(b)).

Analysis of the alizarin red staining quantified by extraction and measurement of absorption at 600 nm revealed significant elevated levels of calcium deposits in all groups containing human serum except serum from D5 (HS: 2.44 ± 0.27, *p* ≤ 0.01; D1: 2.62 ± 0.46, *p* ≤ 0.01; D5: 2.17 ± 0.54, *p* > 0.05; D10: 2.53 ± 0.35, *p* ≤ 0.01) compared to the FCS group (1.30 ± 0.38, Figure 6(b)). No statistically significant difference between the osteogenic differentiations of hBMSCs cultured with human serum from polytrauma patients or with human serum from healthy controls was found (*p* > 0.05, Figure 6(c)).

### 3.7. Chondrogenic Differentiation

**Figure 7:**
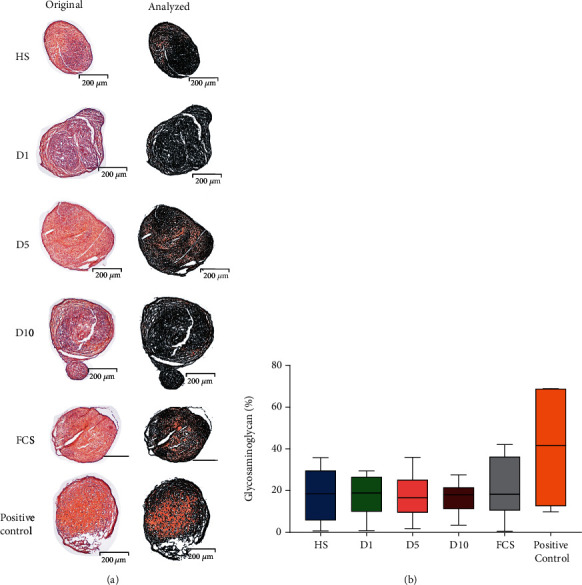
Chondrogenic differentiation potential. (a) Chondrogenic differentiation after 21 days of culture in chondrogenic induction medium containing either HS, D1, D5, D10 serum, FCS, or serum-free (positive control). Images were taken at a magnification of 40x. Glycosaminoglycan content is depicted in orange. Demarcation of areas not stained positively for glycosaminoglycans in black using visual analysis software (based on OpenCV library version 4.1.0). Standard parameters were used in the analysis of each image. (b) Quantitative analysis of the glycosaminoglycan content of the chondrogenic pellet showed no significant differences between different groups (*p* > 0.05, *n* = 4, *N* = 2).

Chondrogenic differentiation with serum-free medium served as positive control in this experiment. The experimental data were nonparametric distributed, and the Kruskal-Wallis test with the Bonferroni correction was used for statistical analysis. All serum groups showed comparable levels of glycosaminoglycans in the extracellular matrix (Figure 7(a)). In the healthy serum group (19.38 ± 11.86%), as well as in D1 (14.61 ± 10.31%), D5 (19.36 ± 10.67%), and D10 (14.36 ± 6.39%), no significant difference in chondrogenic differentiation was seen compared to the FCS group (20.65 ± 13.78%; Figure 7(b)). The same is true for the positive control group (40.46 ± 21.92%) which was serum-free (*p* > 0.05, Figure 7(b)).

## 4. Discussion

Trauma is one of today's major causes of human death and disabilities [37], and young adults are the largest demographic, correlated with social interactions and exposure to labor [38, 39], which made it a heavy burden for the society [40, 41]. As been defined in terms of a high ISS score (≥16) and described as a condition of “severely injured” or “multiple traumata,” polytrauma has a high mortality rate and a high incidence of impairment.

A dysfunctional immune system caused by polytrauma often results in death from sepsis and septic shock or MODS [42–44] by breaking the balance between the systemic inflammatory response syndrome (SIRS) and the compensatory anti-inflammatory response syndrome (CARS) [43, 45, 46]. This imbalance in turn can develop into persistent inflammatory, immunosuppressed, catabolic syndrome (PICS) [47] in the late stage after trauma. Trauma triggers the production of different inflammatory mediators and associated peptides. TNF-*α*, IL-1, IL-6, IL-8, and IL-10 can be secreted from monocytes/macrophages [12, 13], whereby inflammatory cascade activation [10] plays a major role in the development of immune dysfunction and multiple organ dysfunction following polytrauma.

MSC can be triggered by a signal to exhibit immunomodulatory properties, and the most powerful promoters are IFN-*γ*, TNF-*α*, and IL-1*β* [17, 18]. Various adhesion molecules including integrins and selectins are expressed by MSC to regulate the adhesion of the cells to the target tissue's extracellular matrix (ECM) [19, 48]. It is known that MSC can be induced to differentiate into osteoblasts *in vitro* [21] and chondrocytes [22, 23] by culturing in a special differentiation media *in vitro* using classic pellet culture methods. Therefore, the hBMSCs are promising in the treatment of patients suffering from polytrauma, but as it is known that the complex changes of serum cytokines after polytrauma [31] exert a various impact on the characteristics and properties of MSCs [30, 32], it is necessary to explore the cytological effects on hBMSCs at different time points postpolytrauma. Such a study could lead to more knowledge of whether the factor polytrauma has a negative effect on hBMSCs and at which time points hBMSCs should be used to treat polytrauma. Other studies [32, 49] used a single cytokine or stimulated cocktail including multiple factors to answer this question, but these *in vitro* cocktails cannot completely show the serological effects of polytrauma. Therefore, in our study, real serum from patients with polytrauma at D1, D5, and D10 compared to healthy serum was used.

Analysis of the serum cytokine concentration in our study showed that the proinflammatory factors IL-6 and IL-8 and the anti-inflammatory IL-10 were increased on D1 after the induction of trauma. In contrast, other proinflammatory (e.g., IL-1*β*, IL-12, TNF-*α*, and IFN-*γ*) and anti-inflammatory factors (e.g., IL-4, IL-5, and IL-13) were decreased after trauma induction at all time points compared to the serum from healthy controls and with concentrations that slightly recovered from D1 to D10. To sum up these findings, a sharp increase in specific cytokine concentrations was seen after acute traumatic stress as reflected by the D1 group, which decreased close to normal levels following D5 and D10 after polytrauma. In the subsequent D5 and D10 groups, proinflammatory and anti-inflammatory factors are in a relatively balanced state, as their concentrations are comparable to the HS group and showed a fluctuation trend after trauma to maintain a long-term balance which is in line with the theory of SIRS/CARS balancing, as well as the latest PICS theory [50]. A comparable study was carried out by Halbgebauer et al. [51] which conducted a cytokine analysis from seven polytrauma patients (ISS ≥ 32, six males and one female) four hours and one and five days posttrauma and treated the whole blood with or without lipopolysaccharide (LPS). In the results of Halbgebauer et al., the proinflammatory factors IL-12 and IFN-*γ* were downregulated after trauma in D1 when compared with the healthy group, and an upward trend was discovered from D1 to D10. The anti-inflammatory IL-10 was dramatically increased after trauma in D1 in comparison to the healthy group and decreased to a level which was slightly higher than the healthy group in D5, with a rising trend in D10. Upon these cytokines, the results were in line with the findings of this study. However, for IL-4, IL-5, and IL-13, Halbgebauer et al. observed an upregulation in D1 and D5 relative to the healthy group while these cytokines were downregulated in this experiment when compared with HS. Volpin et al. [52] detected the serum cytokines in 58 severe trauma patients (AIS: 3.44 ± 0.5, 35 males, 23 females) several hours after trauma compared to a healthy control group. In the results of Volpin et al., it showed that IL-4 was decreased and IL-6 and IL-8 were increased compared to healthy control as in this study, but it showed a different trend as IL-1*β*, IL-12, and IFN-*γ* were increased when compared with the healthy control, and in this study, a decreased trend was shown for these cytokines compared to HS. Differences in the results from the study by Halbgebauer et al. and Volpin et al. to this study could be explained by different patient groups (age, gender) and the severity of the trauma. At the same time, the half-life of some cytokines is relatively short. For example, TNF-*α* has a half-life of less than 20 minutes and IL-1 only 6 minutes [31]. Therefore, the choice of cytokine detection time point also determines the difference in cytokine changes.

In this study, the proliferation capacities of hBMSCs incubated with serum from D5 polytrauma patients were significantly reduced in comparison to cells incubated with serum from healthy controls. And when using serum from D10, however, the proliferation capacity of hBMSCs was comparable to the control. In accordance with this, the proportion of dead cells upon culture with serum from D5 was significantly higher than that of cells cultured with D1 serum. Based on the results of the cytokine analysis, it could be hypothesized that these effects might be caused by the combined action of multiple cytokines. Among the factors that promote cell proliferation and inhibit apoptosis, EGF can promote the proliferation and migration of MSCs [53, 54], short-term exposure to HGF can improve the survival rate of MSCs [55], and FGF-4 has mitotic activity and can effectively promote the proliferation of MSCs [56]. PDGF-BB can protect cells from apoptosis and is known to regulate cell proliferation [57, 58], and it also has the effect of promoting MSC osteogenic differentiation [59], while IGFBP-4 promotes senescence and apoptosis in MSCs [60]. Most of the above-mentioned factors that promote cell proliferation were significantly reduced in D5 serum, especially FGF-4, while IGFBP-4, which promotes cell senescence and apoptosis, was the highest in serum from D5. EGF and HGF might have a positive effect on proliferation and migration of hBMSCs because their concentrations were higher in serum from polytrauma patients than in the serum from healthy controls. The fact that concentrations of these cytokines were lower in D5 than D1 or D10 might explain the decreased proliferation rate and increased proportion of dead hBMSCs after culture with the respective serum. Therefore, the increase in the concentration of cytokines that can promote cell proliferation and viability is not enough to offset the effects of some cytokines that inhibit cell proliferation and promote cell apoptosis. This may also cause the decreased proliferation and viability.

Amann et al. [32] conducted an experiment where the effect of selected trauma-related factors to the effect of polytrauma serum on the inflammatory response of hBMSCs was compared. Serum from four polytrauma patients was obtained at 0, 4, 12, 24, 48, 120, and 240 hours after their admission to the clinic. The serum of five patients was used to make a medium with a 20% serum concentration to investigate the *in vitro* proliferation of human MSCs after seven days in culture. Proliferation of MSCs was decreased after treatment with serum samples from polytrauma starting after 12 hours up to 240 hours. Compared to this study, the same reduction in the proliferation ability after 72 hours of cultivation time with serum taken at D5 (120 hours) was found. Importantly, Amann et al. determined that different mixtures of cytokines together with serum from healthy controls were not able to induce a similar effect on hBMSC properties as serum from polytrauma patients. This suggests that the effect of polytrauma serum on the proliferation, vitality, and cytotoxicity of MSCs cannot be mimicked by the influence of a single cytokine or even the combination of a few cytokines.

In the CFU-F assay of this study, the results showed that the CFU-F number was not reduced when culturing hBMSCs with polytrauma serum (D1, D5, and D10) as compared to HS. However, the blue staining area of colonies in the polytrauma groups (D1, D5, and D10) was less than that in HS (Figure 2(a)) in visual, especially in the D5 group. With reference to the results of cell proliferation experiments, on the 5^th^ day after trauma, the total number of cells decreased and the proportion of cell death increased, but the ability of colony forming was not reduced, which might lead to less area of staining of CFU-F. The effects of cytokines differentially influencing cell proliferation, cell survival rate, and cell apoptosis are integrated and together mediate the changes in MSCs. And the reduction of factors that promote cell proliferation and the promotion of cell senescence and apoptosis may be related to this result.

The polytrauma serum has no obvious effect on the viability and cytotoxicity of MSC within 24 hours detected by WST assay. Possibly, 24-hour cultivation was not long enough to induce a reduction in cell viability, since a significant increased rate of dead cells was observed after 72 hours of cultivation by using total cell counting and live and dead assay.

Moreover, it was found in this study that there was no significant influence of the different sera on the migration properties of hBMSCs in a transwell migration assay. IGF-1, GRO, and MCP-1 can effectively promote the migration of MSCs [61, 62]. In this study, it was found that the levels of IGF-1 and GRO decreased in the sera from all polytrauma groups. However, the level of MCP-1 was found to be increased, which might result in the zero net effect of the polytrauma sera on the migration properties of hBMSCs. In difference, Hengartner et al. [49] found a promigratory effect of a polytrauma cocktail on MSCs. The method Hengartner et al. used was similar to the transwell assay in this study, and the difference was Hengartner et al. used a mixture of cytokines (cocktail). Combined with the suggestions from the study of Amann et al. [32], these differences strengthen the hypothesis of this study that a mixture of cytokines may not adequately reflect the complex influence of real polytrauma serum on MSCs.

In this study, no significant differences were found in the scratch assay between different groups and different time points. The application of serum-free medium not only ensures that all cells used in the experiment were in P_3_ but also avoids the influence of cell proliferation on migration. It is known that VEGF parasecreted by MSC can promote the migration to repair vascular endothelium; both VEGF [63] and PDGF-BB [64] have the ability to migrate and repair the wounded tissue. In the results of this experiment, the ability of migration in scratch assay did not change according to the trend of cytokines. However, as the scratch assay is an *in vitro* surrogate marker for migration to repair, the actual migration-wound healing ability of hBMSCs *in vivo* may change. Therefore, further exploration *in vivo* is needed.

*In vitro* bone formation in this study was evaluated with alizarin red staining of calcium deposits [65] as well as with alizarin red absorbance measurement at 600 nm [66]. Comparable results were obtained with both methods, and no significant difference in osteogenic differentiation ability of hBMSCs was observed between the HS and polytrauma groups.

Interestingly, in the results of alizarin red absorbance measurement at 600 nm except for the D5 group, all groups containing human serum had a significant higher osteogenic capacity than the FCS group. Perhaps, this is related to the fact that human serum contains more proteins or growth factors [67], and the use of allogeneic human serum MSC culture has a higher beneficial influence on cell proliferation than FCS [68]. In this study, increased serum concentrations of osteopontin, osteoprotegerin, and FGF-9 as well as decreased TIMP-1 concentration were observed. Osteoprotegerin, osteopontin, and FGF-9 play a positive regulatory role in promoting osteogenic differentiation. While osteopontin can effectively promote MSC osteoblastic differentiation [69] and osteoprotegerin inhibits bone resorption [70], FGF-9 effectively increases bone differentiation [71] and is indispensable in long bone repair. In contrast to this, TIMP-1 negatively regulates osteogenic differentiation by attenuating the expression of Runx2 during bone differentiation and also inhibits cell proliferation [72]. This may explain why no negative impact of the polytrauma sera on osteogenic differentiation of hBMSCs was found.

In contrast to the results of this study, Thaweesapphithak et al. [68] conducted osteogenic differentiation experiments of human placenta and umbilical cord-derived MSCs using osteogenic differentiation medium containing human serum for four weeks and found that osteogenic differentiation was not differentially influenced by human serum in comparison to FCS. Popov et al. [73] conducted research on hBMSCs to compare osteogenic differentiation medium containing FCS and human serum, over a differentiation time of 21 days. In the study of Popov et al., human serum was only added to the differentiation medium during the first five days, because Popov et al. found in prior experiments that using human serum resulted only for five days in a better osteogenic differentiation in contrast to the continuous supplementation with human serum. Popov et al. concluded that, in comparison to FCS, the use of human serum enhanced the osteogenic differentiation ability of MSCs and the results are in line with the results obtained in this study.

Glycosaminoglycan staining by safranin O is a reliable method for the quantification of cartilage tissue [74] which was used in this study. The results showed that the chondrogenic differentiation ability of the positive control group (serum-free) was higher than that of the other experimental groups (10% serum added), although there were no significant differences between the experimental groups, indicating that polytrauma serum from all time points had no significant effect on the chondrogenic differentiation of hBMSCs. Proteins from the TGF-*β* family can promote chondrogenic differentiation in MSCs [75, 76]. TGF-*β*1 cooperates with IGF-1 to promote MSC migration and chondrogenic differentiation [77, 78], while IL-1*β* [79] can inhibit chondrogenic differentiation. In the multicytokine array, polytrauma caused decreased concentrations of TGF-*β*1, TGF-*β*2, and TGF-*β*3, especially one day after the trauma, but this was not reflected by a negative influence of the sera on chondrogenic differentiation of hBMSCs *in vitro*. Since TGF-*β*3 was added to the chondrogenic differentiation medium in this experiment, the slight decline of TGF in polytrauma sera might be overruled by the general addition of TGF-*β*3 to the differentiation medium, thereby hiding a possible effect of this reduction.

The following limitations were present in this study. The results were obtained using hBMSCs from only four different donors. Therefore, future studies using hBMSCs from more donors should be conducted to confirm the results found here. Furthermore, pooled sera from patients with polytrauma were used to provide enough volume to perform the experiments. A disadvantage to use pooled serum is that a statistical analysis to prove the difference between groups cannot be performed. However, the most important advantage of using serum from more than 80 polytrauma patients is that individual differences with respect to cytokine changes in serum will likely be zeroed out, and it showed the real concentration change of the cytokines. Furthermore, the polytrauma pooled serum and the pooled serum from healthy donors were matched with respect to age and gender to reduce the influence of these two factors. Additionally, allogeneic serum was used on hBMSCs in this study, and this may lead to proliferation stagnation and death.

At present, many clinical trials using MSCs to treat human diseases can be found on clinicaltrials.gov [80], but the application of MSC to treat human polytrauma is still in the early stage of development. In studies concerning bones and joints, a combination of MSCs and biomaterial scaffolds has been used locally, and its effectiveness has been verified in animal experiments [24–26, 28, 29]. In this study, considering the reconstruction of surgical functions in patients with polytrauma after the basic vital signs have stabilized, the local application of MSCs is a point of interest, for instance, to add BMSC locally into the fracture gap during the final reconstruction of bones and cartilage postpolytrauma, and the influence of serology on MSCs still needs to be considered.

The central idea of the “Damage Control Orthopedics (DCO)” [81] is to implement life-saving procedures early, temporarily stabilize damaged tissues, and finally deal with fracture fixation and osteochondral treatment related to the injury. Because of the imbalance of the SIRS/CARS system, the “first hit” caused by trauma is likely to make the patient face the potential risk of deterioration after the “second hit” of the operation [82, 83]. According to reports, the definitive osteosynthesis on the second to fourth day after trauma increases the chance of MODS [84], and it is beneficial to perform the final fixation within 15 days after trauma to avoid infection of temporary external fixation [85], especially during the 5^th^ to 10^th^ days posttrauma which period has been defined as “window of opportunity” [86]. Based on this knowledge and the results of this study, it could be very promising to add hBMSCs locally into the fracture gap of bones and cartilage during the final reconstructive operation in the “window of opportunity” due to the “DCO” concept. However, a systemic application of MSCs after polytrauma is also conceivable in some studies. In an animal experiment of systemic application of MSCs, Tanrıverdi et al. [87] used allogeneic MSCs which significantly improved the healing ability of the liver and bone in rats after polytrauma. Furthermore, Amann et al. [88] showed that allogeneic MSCs significantly reduced injury score 24 hours after severe blunt chest trauma (TxT). However, previous studies also have shown that systemic application of MSCs, such as systemic intravenous injection, tends to accumulate in the lungs [89] or distribute in other organs, such as the liver, kidneys, long bones, and spleen, 24-48 hours after injection [90]. In addition, it is not easy to detect the donor's MSCs in the whole body after 8-10 days of injection [91]. Furthermore, a study by von Bahr et al. showed that the ability of MSCs to enter the bone marrow was very limited by intravenous injection of MSCs [92]. Therefore, a local administration of MSCs seems more promising to us than a systemic injection.

Altogether, the results indicate that hBMSCs may be useful in the treatment of fractures and cartilage defects of patients suffering from polytrauma, since the *in vitro* regenerative potential of hBMSCs is not negatively affected by cytokines present in serum one, five, or ten days after polytrauma. The best time point for the application of hBMSCs might be at least after five days of polytrauma to overcome the negative effect of polytrauma serum on hBMSCs which were seen at D5. This setup would be in line with the “DCO” concept which consisted of the provisional immobilization of long bone fractures to achieve the advantages of early treatment and minimization of complication risks, such as fat embolism, pathological inflammatory response or severe hemorrhage triggering the lethal triad, and the traumatic effects of major surgery on a patient who is already traumatized (the “second hit” effect) [93].

This study tried to mimic the effects of polytrauma on hBMSCs *in vitro*, but it must be kept in mind that the *in vivo* environment is much more complex, and the *in vitro* conditions might have different effects on the cells than *in vivo*. Therefore, although the results of this study have a certain reference value for clinical applications, further *in vivo* verification is necessary.

## 5. Conclusions

The composition of polytrauma serum changes over time after the trauma incident. Therefore, the effect on hBMSC biology is differentially affected by sera obtained from polytrauma patients at different time intervals after trauma. Prolonged exposure to polytrauma serum collected on D5 exerts a negative effect on hBMSCs with reduced proliferation ability and a higher cell death ratio. Polytrauma serum did not significantly affect the colony forming ability, cells' migration, and scratch healing ability, and no significant effects on the ability to differentiate into bone and cartilage tissues were found.

## Figures and Tables

**Table 1:** List of donors.

Parameters	PT patients	HS volunteers	hBMSC donors	*p* value
Age (Y)	45 (29.5-55)	39 (31.5-53)	43 (41.5-56.25)	0.69
Gender (M/F)	56/28	2/1	3/1	0.94
ISS score	34 (27-43.5)	n/a	n/a	

Note: parameters are given as median (interquartile range) as the data are nonparametric, which was tested by the Shapiro-Wilk test. The age (in years, Y) and gender (male/female, M/F) were compared by the Kruskal-Wallis test and showed no statistical differences (*p* > 0.05).

## Data Availability

The data used to support the findings of this study are available from the corresponding author upon request.

## Supplementary Materials

Supplementary Materials Supplementary Figure S1: the level change of cytokines: growth factors (EGF, HGF, FGF-4, and IGFBP-4) and migration factors (IGF-1, GRO-*α*, MCP-1, VEGF, and PDGF-BB).

## References

[1] Sakran J. V., Greer S. E., Werlin E., McCunn M. (2012). Care of the injured worldwide: trauma still the neglected disease of modern society. *Scandinavian Journal of Trauma, Resuscitation and Emergency Medicine*.

[2] Unfallchirurgie D. G. F. Definition of polytrauma. https://www.dgu-online.de/patienten/haeufige-diagnosen/schwerverletzte/polytrauma.html.

[3] Frenzel S., Krenn P., Heinz T., Negrin L. L. (2017). Does the applied polytrauma definition notably influence outcome and patient population? - a retrospective analysis. *Scandinavian Journal of Trauma, Resuscitation and Emergency Medicine*.

[4] Baker S. P., O'Neill B. (1976). The injury severity score: an update. *The Journal of Trauma*.

[5] Baker S. P., Oʼneill B., Haddon W., Long W. B. (1974). The injury severity SCORE. *The Journal of Trauma*.

[6] Marzi I., Lustenberger T., Störmann P., Mörs K., Wagner N., Wutzler S. (2019). Increasing overhead ressources of the trauma room. *Unfallchirurg*.

[7] de Vries R., Reininga I. H. F., Pieske O., Lefering R., el Moumni M., Wendt K. (2018). Injury mechanisms, patterns and outcomes of older polytrauma patients-an analysis of the Dutch Trauma Registry. *PLoS One*.

[8] Pape H. C., Lefering R., Butcher N. (2014). The definition of polytrauma revisited. *Journal of Trauma and Acute Care Surgery*.

[9] Ciriello V., Gudipati S., Stavrou P. Z., Kanakaris N. K., Bellamy M. C., Giannoudis P. V. (2013). Biomarkers predicting sepsis in polytrauma patients: current evidence. *Injury*.

[10] Keel M., Trentz O. (2005). Pathophysiology of polytrauma. *Injury*.

[11] Fabiano G., Pezzolla A., Filograna M. A., Ferrarese F. (2008). Traumatic shock--physiopathologic aspects. *Il Giornale di Chirurgia*.

[12] Lenz A., Franklin G. A., Cheadle W. G. (2007). Systemic inflammation after trauma. *Injury*.

[13] Toft P., Andersen S. K., Tønnesen E. K. (2003). The systematic inflammatory response after major trauma. *Ugeskrift for Laeger*.

[14] Trancă S. D., Petrişor C. L., Hagău N. (2014). Biomarkers in polytrauma induced systemic inflammatory response syndrome and sepsis - a narrative review. *Romanian journal of anaesthesia and intensive care*.

[15] Ullah I., Subbarao R. B., Rho G. J. (2015). Human mesenchymal stem cells - current trends and future prospective. *Bioscience Reports*.

[16] Zha K., Li X., Yang Z. (2021). Heterogeneity of mesenchymal stem cells in cartilage regeneration: from characterization to application. npj. *Regenerative Medicine*.

[17] Krampera M. (2011). Mesenchymal stromal cell ‘licensing’: a multistep process. *Leukemia*.

[18] Mougiakakos D., Jitschin R., Johansson C. C., Okita R., Kiessling R., le Blanc K. (2011). The impact of inflammatory licensing on heme oxygenase-1-mediated induction of regulatory T cells by human mesenchymal stem cells. *Blood*.

[19] Sohni A., Verfaillie C. M. (2013). Mesenchymal stem cells migration homing and tracking. *Stem Cells International*.

[20] Eder C., Schmidt-Bleek K., Geissler S. (2020). Mesenchymal stromal cell and bone marrow concentrate therapies for musculoskeletal indications: a concise review of current literature. *Molecular Biology Reports*.

[21] Friedenstein A. J., Chailakhyan R. K., Gerasimov U. V. (1987). Bone marrow osteogenic stem cells: in vitro cultivation and transplantation in diffusion chambers. *Cell and Tissue Kinetics*.

[22] Ashton B. A., Allen T. D., Howlett C. R., Eaglesom C. C., Hattori A., Owen M. (1980). Formation of bone and cartilage by marrow stromal cells in diffusion chambers in vivo. *Clinical Orthopaedics and Related Research*.

[23] Beresford J. N. (1989). Osteogenic stem cells and the stromal system of bone and marrow. *Clinical Orthopaedics and Related Research*.

[24] Arinzeh T. L. (2005). Mesenchymal stem cells for bone repair: preclinical studies and potential orthopedic applications. *Foot and Ankle Clinics*.

[25] Duan W., Chen C., Haque M., Hayes D., Lopez M. J. (2018). Polymer-mineral scaffold augments in vivo equine multipotent stromal cell osteogenesis. *Stem Cell Research & Therapy*.

[26] Sun H., Qu Z., Guo Y., Zang G., Yang B. (2007). In vitro and in vivo effects of rat kidney vascular endothelial cells on osteogenesis of rat bone marrow mesenchymal stem cells growing on polylactide-glycoli acid (PLGA) scaffolds. *Biomedical Engineering Online*.

[27] Nejadnik H., Diecke S., Lenkov O. D. (2015). Improved approach for chondrogenic differentiation of human induced pluripotent stem cells. *Stem Cell Reviews and Reports*.

[28] Yang J., Zhang Y. S., Yue K., Khademhosseini A. (2017). Cell-laden hydrogels for osteochondral and cartilage tissue engineering. *Acta Biomaterialia*.

[29] Man Z., Yin L., Shao Z. (2014). The effects of co-delivery of BMSC-affinity peptide and rhTGF-*β*1 from coaxial electrospun scaffolds on chondrogenic differentiation. *Biomaterials*.

[30] Huber-Lang M., Wiegner R., Lampl L., Brenner R. E. (2016). Mesenchymal stem cells after polytrauma: actor and target. *Stem Cells International*.

[31] DeLong W. G., Born C. T. (2004). Cytokines in patients with polytrauma. *Clinical Orthopaedics and Related Research*.

[32] Amann E., Groß A., Rojewski M. T. (2019). Inflammatory response of mesenchymal stromal cells after in vivo exposure with selected trauma-related factors and polytrauma serum. *PLoS One*.

[33] Wiegner R., Rudhart N. E., Barth E. (2018). Mesenchymal stem cells in peripheral blood of severely injured patients. *European Journal of Trauma and Emergency Surgery*.

[34] RayBio RayBio® Human Cytokine Antibody Array G-Series 5-Cat# AAH-CYT-G5-8. 2014. http://www.raybiotech.com/files/manual/Antibody-Array/AAH-CYT-G5.pdf.

[35] Bradski G. (2000). The OpenCV Library. *Dr. Dobb's Journal of Software Tools*.

[36] Morpheus https://software.broadinstitute.org/morpheus.

[37] al-Thani H., el-Menyar A., Asim M. (2019). Evolution of the Qatar trauma system: the journey from inception to verification. *Journal of Emergencies, Trauma, and Shock*.

[38] Wang J., Lu H., Sun Z., Wang T. (2020). Exploring factors influencing injury severity of vehicle at-fault accidents: a comparative analysis of passenger and freight vehicles. *International Journal of Environmental Research and Public Health*.

[39] Prevention, CCfDCa Saving lives and protecting people from violence and injuries. https://www.cdc.gov/about/pdf/cdc-recent-accomplishments.pdf.

[40] van Beeck E. F., van Roijen L., Mackenbach J. P. (1997). Medical costs and economic production losses due to injuries in the Netherlands. *The Journal of Trauma*.

[41] Bastida J. L., Aguilar P. S., González B. D. (2004). The economic costs of traffic accidents in Spain. *The Journal of Trauma*.

[42] Geneva W. H. O. The global burden of disease. https://www.who.int/healthinfo/global_burden_disease/en/.

[43] Wutzler S., Lustenberger T., Relja B., Lehnert M., Marzi I. (2013). Pathophysiologie des Polytraumas. *Chirurg*.

[44] Wafaisade A., Lefering R., Bouillon B. (2011). Epidemiology and risk factors of sepsis after multiple trauma: an analysis of 29,829 patients from the Trauma Registry of the German Society for Trauma Surgery. *Critical Care Medicine*.

[45] Adib-Conquy M., Cavaillon J. M. (2009). Compensatory anti-inflammatory response syndrome. *Thrombosis and Haemostasis*.

[46] Sturm R., Xanthopoulos L., Heftrig D. (2020). Regulatory T cells modulate CD4 proliferation after severe trauma via IL-10. *Journal of Clinical Medicine*.

[47] Mira J. C., Brakenridge S. C., Moldawer L. L., Moore F. A. (2017). Persistent inflammation, immunosuppression and catabolism syndrome. *Critical Care Clinics*.

[48] Ren G., Zhao X., Zhang L. (2010). Inflammatory cytokine-induced intercellular adhesion molecule-1 and vascular cell adhesion molecule-1 in mesenchymal stem cells are critical for immunosuppression. *Journal of Immunology*.

[49] Hengartner N.-E., Fiedler J., Schrezenmeier H., Huber-Lang M., Brenner R. E. (2015). Crucial role of IL1beta and C3a in the in vitro-response of multipotent mesenchymal stromal cells to inflammatory mediators of polytrauma. *PLoS One*.

[50] Binkowska A. M., Michalak G., Słotwiński R. (2015). Current views on the mechanisms of immune responses to trauma and infection. *Central-European Journal of Immunology*.

[51] Halbgebauer R., Kellermann S., Schäfer F. (2020). Functional immune monitoring in severely injured patients-a pilot study. *Scandinavian Journal of Immunology*.

[52] Volpin G., Cohen M., Assaf M., Meir T., Katz R., Pollack S. (2014). Cytokine levels (IL-4, IL-6, IL-8 and TGF*β*) as potential biomarkers of systemic inflammatory response in trauma patients. *International Orthopaedics*.

[53] Tamama K., Fan V. H., Griffith L. G., Blair H. C., Wells A. (2006). Epidermal growth factor as a candidate for ex vivo expansion of bone marrow-derived mesenchymal stem cells. *Stem Cells*.

[54] Knight C., James S., Kuntin D. (2019). Epidermal growth factor can signal via *β*-catenin to control proliferation of mesenchymal stem cells independently of canonical Wnt signalling. *Cellular Signalling*.

[55] Forte G., Minieri M., Cossa P. (2006). Hepatocyte growth factor effects on mesenchymal stem cells: proliferation, migration, and differentiation. *Stem Cells*.

[56] Farré J., Farré J., Roura S. (2007). FGF-4 increases in vitro expansion rate of human adult bone marrow-derived mesenchymal stem cells. *Growth Factors*.

[57] Zhang J.-M., Feng F. E., Wang Q. M. (2016). Platelet-derived growth factor-BB protects mesenchymal stem cells (MSCs) derived from immune thrombocytopenia patients against apoptosis and senescence and maintains MSC-mediated immunosuppression. *Stem Cells Translational Medicine*.

[58] Lennon D., Solchaga L. A., Somoza R. A., Schluchter M. D., Margevicius S., Caplan A. I. (2018). Human and rat bone marrow-derived mesenchymal stem cells differ in their response to fibroblast growth factor and platelet-derived growth factor. *Tissue Engineering. Part A*.

[59] Hung B. P., Hutton D. L., Kozielski K. L. (2015). Platelet-derived growth factor BB enhances osteogenesis of adipose-derived but not bone marrow-derived mesenchymal stromal/stem cells. *Stem cells (Dayton, Ohio)*.

[60] Severino V., Alessio N., Farina A. (2013). Insulin-like growth factor binding proteins 4 and 7 released by senescent cells promote premature senescence in mesenchymal stem cells. *Cell Death & Disease*.

[61] Ponte A. L., Marais E., Gallay N. (2007). The in vitro migration capacity of human bone marrow mesenchymal stem cells: comparison of chemokine and growth factor chemotactic activities. *Stem Cells*.

[62] Bayo J., Real A., Fiore E. J. (2017). IL-8, GRO and MCP-1 produced by hepatocellular carcinoma microenvironment determine the migratory capacity of human bone marrow-derived mesenchymal stromal cells without affecting tumor aggressiveness. *Oncotarget*.

[63] Barrientos S., Stojadinovic O., Golinko M. S., Brem H., Tomic-Canic M. (2008). Perspective article: growth factors and cytokines in wound healing. *Wound Repair and Regeneration*.

[64] Ball S. G., Shuttleworth C. A., Kielty C. M. (2007). Mesenchymal stem cells and neovascularization: role of platelet-derived growth factor receptors. *Journal of Cellular and Molecular Medicine*.

[65] Puchtler H., Meloan S. N., Terry M. S. (1969). On the history and mechanism of alizarin and alizarin red S stains for calcium. *The Journal of Histochemistry and Cytochemistry*.

[66] Hanna H., Mir L. M., Andre F. M. (2018). In vitro osteoblastic differentiation of mesenchymal stem cells generates cell layers with distinct properties. *Stem Cell Research & Therapy*.

[67] Tekkatte C., Gunasingh G. P., Cherian K. M., Sankaranarayanan K. (2011). “Humanized” stem cell culture techniques: the animal serum controversy. *Stem Cells International*.

[68] Thaweesapphithak S., Tantrawatpan C., Kheolamai P., Tantikanlayaporn D., Roytrakul S., Manochantr S. (2019). Human serum enhances the proliferative capacity and immunomodulatory property of MSCs derived from human placenta and umbilical cord. *Stem Cell Research & Therapy*.

[69] Carvalho M. S., Poundarik A. A., Cabral J. M. S., da Silva C. L., Vashishth D. (2018). Biomimetic matrices for rapidly forming mineralized bone tissue based on stem cell-mediated osteogenesis. *Scientific Reports*.

[70] Zhou S., Fang X., Xin H., Li W., Qiu H., Guan S. (2013). Osteoprotegerin inhibits calcification of vascular smooth muscle cell via down regulation of the Notch1-RBP-J*κ*/Msx2 signaling pathway. *PLoS One*.

[71] Wallner C., Schira J., Wagner J. M. (2015). Application of VEGFA and FGF-9 enhances angiogenesis, osteogenesis and bone remodeling in type 2 diabetic long bone regeneration. *PLoS One*.

[72] Liang T., Gao W., Zhu L. (2019). TIMP-1 inhibits proliferation and osteogenic differentiation of hBMSCs through Wnt/*β*-catenin signaling. *Bioscience Reports*.

[73] Popov A., Scotchford C., Grant D., Sottile V. (2019). Impact of serum source on human mesenchymal stem cell osteogenic differentiation in culture. *International Journal of Molecular Sciences*.

[74] Király K., Lammi M., Arokoski J. (1996). Safranin O reduces loss of glycosaminoglycans from bovine articular cartilage during histological specimen preparation. *The Histochemical Journal*.

[75] Goude M. C., McDevitt T. C., Temenoff J. S. (2014). Chondroitin sulfate microparticles modulate transforming growth factor-*β*1-induced chondrogenesis of human mesenchymal stem cell spheroids. *Cells, Tissues, Organs*.

[76] Grafe I., Alexander S., Peterson J. R. (2018). TGF-*β* family signaling in mesenchymal differentiation. *Cold Spring Harbor Perspectives in Biology*.

[77] Longobardi L., O'Rear L., Aakula S. (2006). Effect of IGF-I in the chondrogenesis of bone marrow mesenchymal stem cells in the presence or absence of TGF-beta signaling. *Journal of Bone and Mineral Research*.

[78] Fu X., Liu G., Halim A., Ju Y., Luo Q., Song A. G. (2019). Mesenchymal stem cell migration and tissue repair. *Cell*.

[79] Felka T., Schäfer R., Schewe B., Benz K., Aicher W. K. (2009). Hypoxia reduces the inhibitory effect of IL-1*β* on chondrogenic differentiation of FCS-free expanded MSC. *Osteoarthritis and Cartilage*.

[80] Source O. (2021). *Clinical Trials*.

[81] Shapiro M. B., Jenkins D. H., William Schwab C., Rotondo M. F. (2000). Damage control: collective review. *The Journal of Trauma*.

[82] Gebhard F., Huber-Lang M. (2008). PolytraumÅ pathophysiology and management principles. *Langenbeck's Archives of Surgery*.

[83] Giannoudis P. V. (2003). Current concepts of the inflammatory response after major trauma: an update. *Injury*.

[84] Pape H., Stalp M., Griensven M. V., Weinberg A., Dahlweit M., Tscherne H. (1999). Optimaler Zeitpunkt der Sekundäroperation bei polytrauma Eine Evaluation an 4314 Schwerverletzten. *Chirurg*.

[85] Harwood P. J., Giannoudis P. V., Probst C., Krettek C., Pape H. C. (2006). The risk of local infective complications after damage control procedures for femoral shaft fracture. *Journal of Orthopaedic Trauma*.

[86] Stahel P. F., Heyde C. E., Wyrwich W., Ertel W. (2005). Aktuelle konzepte des polytraumamanagements: von ATLS zu damage control. *Der Orthopäde*.

[87] Tanrıverdi A. K., Polat O., Elçin A. E. (2020). Mesenchymal stem cell transplantation in polytrauma: evaluation of bone and liver healing response in an experimental rat model. *European Journal of Trauma and Emergency Surgery*.

[88] Amann E. M., Rojewski M. T., Rodi S. (2018). Systemic recovery and therapeutic effects of transplanted allogenic and xenogenic mesenchymal stromal cells in a rat blunt chest trauma model. *Cytotherapy*.

[89] Fischer U. M., Harting M. T., Jimenez F. (2009). Pulmonary passage is a major obstacle for intravenous stem cell delivery: the pulmonary first-pass effect. *Stem Cells and Development*.

[90] Gao J., Dennis J. E., Muzic R. F., Lundberg M., Caplan A. I. (2001). The dynamic in vivo distribution of bone marrow-derived mesenchymal stem cells after infusion. *Cells, Tissues, Organs*.

[91] Kraitchman D. L., Tatsumi M., Gilson W. D. (2005). Dynamic imaging of allogeneic mesenchymal stem cells trafficking to myocardial infarction. *Circulation*.

[92] von Bahr L., Batsis I., Moll G. (2012). Analysis of tissues following mesenchymal stromal cell therapy in humans indicates limited long-term engraftment and no ectopic tissue formation. *Stem Cells*.

[93] Guerado E., Bertrand M. L., Cano J. R., Cerván A. M., Galán A. (2019). Damage control orthopaedics: state of the art. *World Journal of Orthopedics*.

